# The Evolution of Quorum Sensing as a Mechanism to Infer Kinship

**DOI:** 10.1371/journal.pcbi.1004848

**Published:** 2016-04-27

**Authors:** Jonas Schluter, Armin P. Schoech, Kevin R. Foster, Sara Mitri

**Affiliations:** 1 Computational Biology Program, Sloan-Kettering Institute, Memorial Sloan-Kettering Cancer Center, New York, New York, United States of America; 2 Department of Systems Biology, Harvard Medical School, Boston, Massachusetts, United States of America; 3 Department of Zoology, University of Oxford, Oxford, United Kingdom; 4 Department of Fundamental Microbiology, University of Lausanne, Switzerland; University of Cambridge, UNITED KINGDOM

## Abstract

Bacteria regulate many phenotypes via quorum sensing systems. Quorum sensing is typically thought to evolve because the regulated cooperative phenotypes are only beneficial at certain cell densities. However, quorum sensing systems are also threatened by non-cooperative “cheaters” that may exploit quorum-sensing regulated cooperation, which begs the question of how quorum sensing systems are maintained in nature. Here we study the evolution of quorum sensing using an individual-based model that captures the natural ecology and population structuring of microbial communities. We first recapitulate the two existing observations on quorum sensing evolution: density-dependent benefits favor quorum sensing but competition and cheating will destabilize it. We then model quorum sensing in a dense community like a biofilm, which reveals a novel benefit to quorum sensing that is intrinsically evolutionarily stable. In these communities, competing microbial genotypes gradually segregate over time leading to positive correlation between density and genetic similarity between neighboring cells (relatedness). This enables quorum sensing to track genetic relatedness and ensures that costly cooperative traits are only activated once a cell is safely surrounded by clonemates. We hypothesize that under similar natural conditions, the benefits of quorum sensing will not result from an assessment of density but from the ability to infer kinship.

## Author Summary

Bacteria secrete signal molecules into their environment and use these to regulate many of their key phenotypes. This is called quorum sensing and it is thought to evolve because it allows cells to sense their density. Here we propose a new function for quorum sensing that sheds light on its evolution. We develop a realistic model of a bacterial community and show that quorum sensing can function as a way to outcompete neighbors in patches occupied by many different genotypes. Growing aggressively at first makes quorm sensing genotypes a match for competitors. This strategy allows them to surround themselves with clonemates before reallocating resources to costly traits like cooperative secretions. This works because quorum sensing can act as a timer, which cells can use to infer how related they are to their neighbours and tune their investment into costly and exploitable cooperation based on the threat of competition from unrelated genotypes.

## Introduction

Microbes use quorum sensing to regulate a large number of phenotypes. During growth, cells secrete autoinducers, which are small, diffusible compounds that accumulate in the environment. High autoinducer concentration around cells induces expression of many metabolically costly traits, which includes secretions that promote the growth of surrounding cells [1–6]. The canonical explanation for the function of quorum sensing is that autoinducer concentrations can be used as a proxy for cell density. Estimating the density of cells in a given environment allows microbes to cooperate in a coordinated manner. Cells can tune the expression of density-dependent phenotypes, like virulence factors or secreted enzymes, so that they are only expressed when there are enough cells to make them useful [1, 4, 7, 8]. The benefits of quorum sensing can also be affected by diffusion conditions [9–11], which may favor the evolution of multiple quorum sensing signals [12].

Quorum sensing strains then may outcompete strains that constitutively express cooperative phenotypes. However, this does not guarantee quorum sensing evolution because there is also competition from strains that do not cooperate at all, often known as “cheater” genotypes [4, 13–16]. For example, *Pseudomonas aeruginosa* cells with a defective *lasR* gene preventing them from responding to quorum sensing signals, can outcompete cooperating wild-type cells [4, 14]. This is because the mutants have a higher growth rate since they do not produce the costly cooperative secretions, but can benefit from the secretions of wild-type cells. Quorum sensing then has the potential to be evolutionarily unstable in mixed genotype cultures [4, 7, 8, 13, 16, 17].

Such mixed genotype cultures are expected to be common in microbes, which often live in highly diverse and dense communities where many different genotypes meet and compete [5, 18–21]. What then maintains cooperative phenotypes in microbes? One explanation is the spontaneous formation of clonal patches within microbial communities by cell division (also see [22]). As cells divide and grow in dense and nutrient-limited conditions, bottlenecks occur that cause genotypes to segregate into large clonal patches. The formation of these patches in initially diverse microbial groups is an empirically well-established phenomenon, having been observed in numerous microbial species including bacteria, yeast and amoeba, and different experimental conditions including agar plates and flow cells [23–32]. Theoretical work suggests that this patch formation in microbial colonies can stabilize the use of beneficial secretions because benefitting cells are now highly related [33], which has recently been verified experimentally [34, 35].

Here we investigate the costs and benefits of quorum sensing controlling the secretion of a public good—a key evolutionary dilemma [49]—in diverse and dense microbial communities. We use a realistic individual-based model of microbes that captures key features of the natural ecology of microbial groups [29, 33, 36–39]. We first recapitulate the most cited function of quorum sensing in single genotype groups: it allows cells to respond to cell density and diffusive conditions. We then consider what happens when quorum sensing cells are surrounded by competing genotypes. These competitors can either be cells that constitutively produce beneficial secretions (cooperators) or non-producers that have the potential to act as cheater genotypes. Our analysis shows how quorum sensing can evolve in dense and diverse microbial communities by enabling quorum sensing genotypes to outcompete both types of competitors. The benefit of quorum sensing we observe is not directly associated with the inference of cell density. In dense communities containing many different genotypes, quorum sensing additionally, and critically benefits cells by inferring when they are surrounded by clonemates.

## Results

### Quorum sensing increases performance in clonal groups

According to the canonical view, quorum sensing allows cells to initiate shared beneficial traits only once cells are dense enough for the group to benefit. Shared beneficial traits can take a variety of forms including detoxification, slow growth rate and virulence factors. However, the archetypal shared beneficial trait is a secretion that helps cells acquire nutrients or minerals, like enzymes or chelators [14, 40]. We therefore phrase our analysis in terms of secretions here but the basic conclusions should apply for any trait that carries an energetic cost to the cell that expresses it and benefits surrounding cells.

**Fig 1:**
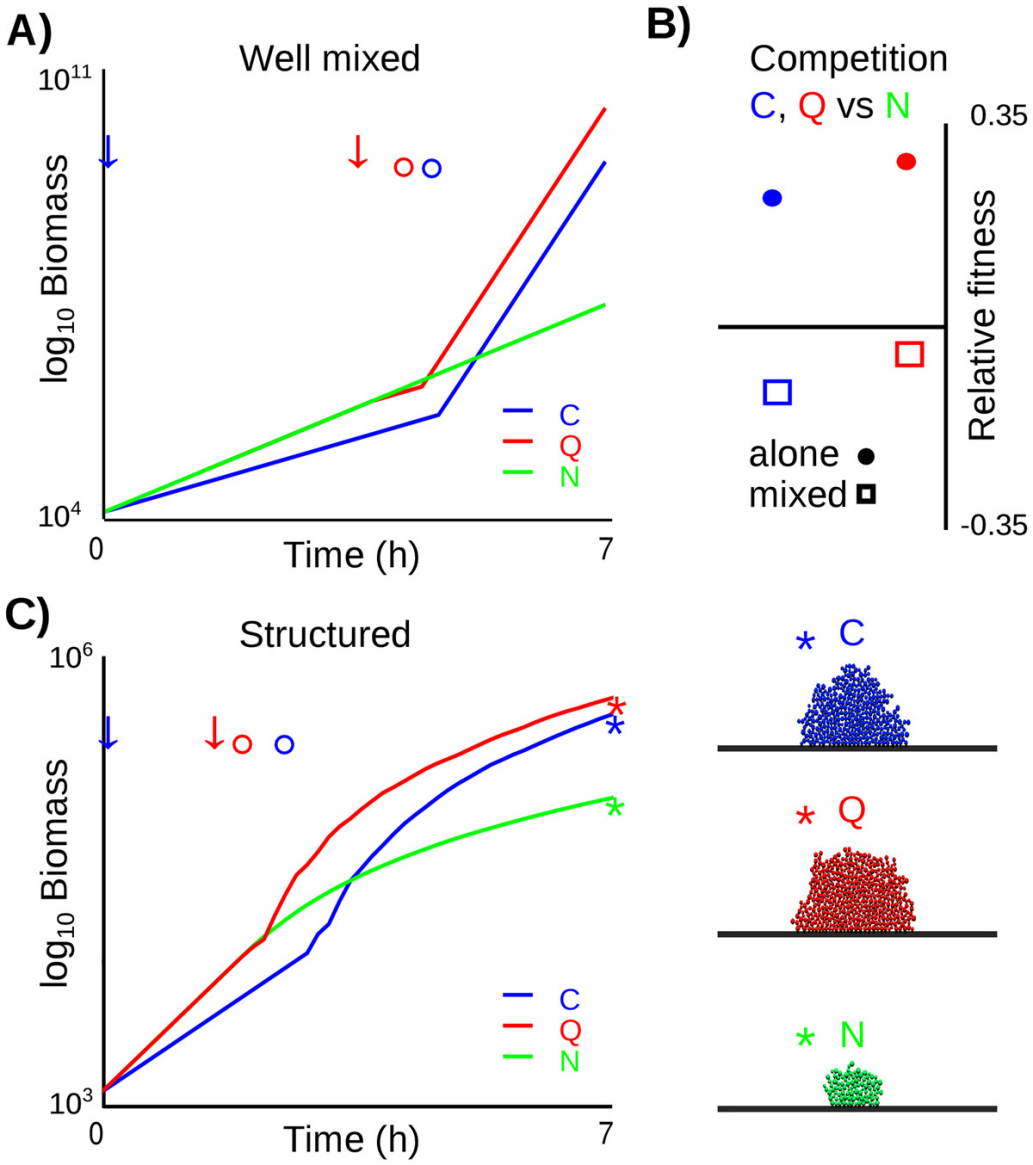
Canonical Quorum Sensing. A) Growth of biomass over time in simulations of well-mixed cultures of a constitutively public good secreting genotype (**C**, blue), a quorum sensing genotype that only begins secreting public goods after a threshold concentration of the autoinducer has been reached (**Q**, red) and a control genotype that does not produce any public goods (**N**, green). The arrows on the time axis indicate the onset of public good secretion by the two secretor genotypes, circles indicate the first time point at which the public good has reached above-threshold concentration in the simulation and cells begin to benefit from the public good. B) Relative fitness of **C** and **Q** (Materials and Methods) compared with competitors of genotype **N** when growing alone (filled circles) or in direct competition in well-mixed culture (open squares). The horizontal line represents equal fitness of **N** and its competitor (either **C** or **Q**). Secretion (**C** and **Q**) is only favoured over non secretion when genotypes grow alone. C) Comparison of biomass growth for the three genotypes in spatially structured clonal colonies. Time axis labels as in A). On the right: snapshots of the simulated colonies at t = 7 h marked with asterisks in the plot.

We first test the prediction that density-dependent benefits will allow quorum sensing cells (**Q**) to have an advantage over both constitutive secretors (**C**) and non-secretors (**N**). Density is defined as mass per unit volume: in our case, we take this volume to be constant (the whole simulation space) and simply quantify the total biomass. Starting from a single cell in a well-mixed, “liquid” environment (Materials and Methods), **N** cells grow and divide, such that the biomass of the population increases exponentially over the course of the simulation (Fig 1, green line). **C** cells, on the other hand, initially grow slower than **N** because a fraction of their growth is redirected into the secretion of a factor that helps all cells around them to grow. For example, this could be a protease that breaks down proteins into amino acids or peptides for import into the cell [14, 40]. After an initial phase of slow growth, **C** cells then experience a burst in growth once the concentration of the secreted factor is high enough to have an effect (Fig 1A, blue line, and Fig 1C). This density-dependent benefit to secretions is consistent with experimental evidence [41–43] and is in accordance with previous models [7, 33, 38].

**Q** cells perform better than both **N** and **C**. In our model, quorum sensing cells secrete a non-costly diffusible autoinducer, sense its concentration, and only begin to produce the costly public good once a threshold concentration of the autoinducer is perceived. Quorum sensing cells grow identically to the **N** cells initially, and only start to produce public goods once they reach quorum, at which point the public good quickly accumulates to the threshold concentration such that all cells benefit (Fig 1A and 1C, solid red lines). These results confirm that our simulations capture the canonical paradigm of quorum sensing, in which **Q** cells can prevent wasteful secretion of public goods and maximise the efficiency of public good secretion. We assume that the cells constantly secrete the autoinducer and do not consider positive feedback in its production, which can sometimes occur [44–46]. Introducing positive feedback would allow an increased potential to tune and optimise the timing of the quorum sensing response. Our predictions on the evolution of quorum sensing, therefore, are conservative in the sense that adding in additional complexity should only improve the scope for quorum sensing to evolve.

### The problem of cheating for the evolution of quorum sensing

Our first model assumes that the different genotypes live alone in clonal communities with no direct interactions between genotypes. This assumption favors strains with the maximum yield of biomass: the more cells a colony can generate, the higher the probability that its offspring will colonize new patches. While distinct clonal patches may well capture the biology of some microbial species, the extreme levels of diversity found in nature [18–20, 47, 48] suggest that strains are often surrounded by other genotypes, whether they are of the same species generated through genetic diversification or of distantly-related species. We next implement such competition in the model and recapitulate previous experimental [4, 22] and theoretical work [7, 10], which shows how quorum sensing can be evolutionarily unstable in genetically diverse and well-mixed environments. Specifically, we next initialize the system with two cells that produce secretions, one cell of either the **C** or **Q** genotype together with a non-secretor cell (**N**), in a well-mixed environment, equivalent to growth in liquid. While both **C** and **Q** outcompete non-secretors in the clonal groups (Fig 1A), direct competition results in cells from all genotypes benefiting from the public good while non-secretors do not incur the costs of its secretion. They therefore outcompete both secretor genotypes (Fig 1B). As predicted by previous theoretical and experimental models [4, 7, 8, 10, 22, 36], then, our simulations demonstrate how secretors can be exploited by non-secretors in well-mixed groups and our model is an example of a public goods dilemma [13, 49].

### Constitutive secretors lose when surrounded by competing genotypes

**Fig 2:**
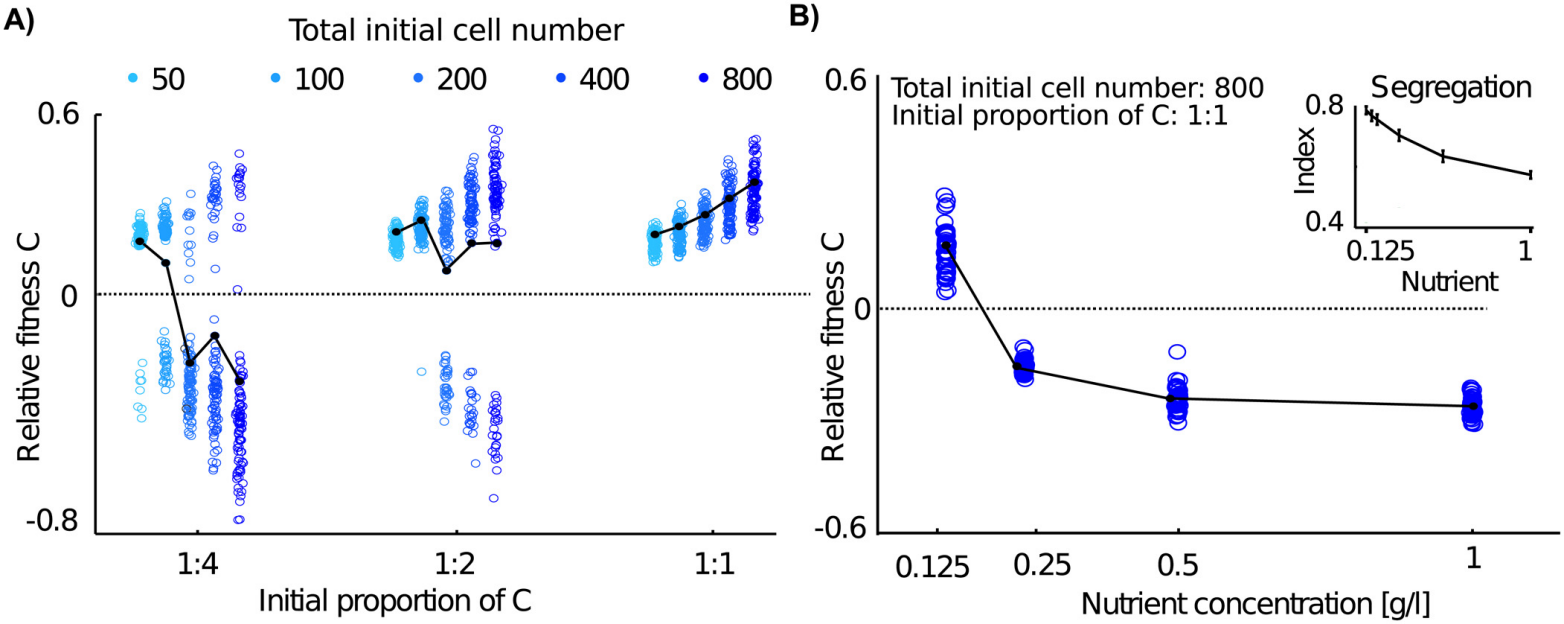
Constitutive secretors lose when surrounded by non-secretor cells. A) Relative fitness of a constitutive secretor genotype **C** in competition with non-secretor genotypes **N** in spatially structured simulations. Constitutive secretors can outcompete non-secretors when competing with few other genotypes (higher proportion of **C** cells) and fewer cells (lower initial cell number). At high evolutionary competition where many non-secretor genotypes compete with the secretor genotype (1:4), constitutive secretors will be outcompeted by non-secretors. The black line connects mean relative fitness values (Materials and Methods), while the dotted line indicates equal fitness of **N** and **C**. B) Constitutive secretion can succeed (relative fitness >0) when genotypes segregate from each other in space. This occurs when nutrients are limited (strong ecological competition). Here, 400 cells of genotype **C** were competed with 400 cells of **N** and nutrient concentrations were varied. Inset: The segregation index at the end of simulations in pure colonies of **N** decreases with nutrient concentrations and the colony remains more mixed. Non-secretors were used for this analysis, because the positive feedback of successful public good cooperation would also increase the segregation index (Materials and Methods).

While direct competition in liquid favors non-secretors, theoretical and experimental work shows that secretor genotypes can outcompete non-secretor genotypes in direct competition in a spatially structured environment [24, 33, 38, 50]. Therefore, we next map out the effects of spatial structure on direct competitions between secretors and non-secretors [33], before then considering how quorum sensing genotypes will fare under the same conditions (Fig 2). Previous work has shown that when nutrients are limiting, secretors can sometimes outcompete non-secretors in direct competition [33]. The reason is that low nutrient levels lead to population bottlenecks and the emergence of large patches of a single genotype, which prevents **N** genotypes from using the secretions of **C** genotypes (Fig 2B, [33–35]). These bottlenecks leading from well-mixed to spatially structured populations represent a well-studied phenomenon that has been widely observed in microbial communities [29, 30, 51, 52]. In social evolution terminology, the population bottlenecks drive an increase over time in genetic relatedness—the probability that two individuals are more genetically similar than the population average, or in this context, the probability that a focal cell is surrounded by genetically identical cells compared to other genotypes (Materials and Methods)—which is one of the major predictors of the evolution of cooperation [33, 53].

However, secretors are often outcompeted by non-secretors, despite bottlenecking effects that increase relatedness. In particular, whenever constitutive secretors start to grow surrounded by cells of competing genotypes (low genetic relatedness, [33, 38]), it is more difficult for them to compete successfully (Fig 2A, 1:4 competitions). Why does a constitutive secretor fare badly when surrounded by non-secretors? The reason is that constitutive secretors are providing secretions that benefit non-secretors as much as secretors but only they are paying the cost to make them. The result is that faster growing non-secretors can physically overgrow and smother them, preventing secretors from capitalizing on the benefits of their cooperation. Constitutive secretors lose then under conditions of nutrient limitation and high diversity, both of which are thought to often occur in natural microbial communities [5, 19, 20, 38, 54]. Indeed, these conditions are expected whenever a focal genotype lands in an environment that has already been populated by other genotypes.

### Quorum sensing is advantageous under strong competition

Our initial analysis shows that when the social environment is highly competitive—nutrients are limiting and there are many genotypes—constitutive secretion can be a disadvantageous strategy. We next explore how a quorum sensing strain performs under such competitive conditions. We competed a **Q** strain, or a **C** strain, in pairwise competitions against the non-secretor **N** strain. Again, our scenario is that the population comprises a number of non-secretor (**N**) strains and we ask: what is the fitness of a single focal secretor of the **Q** or **C** strain, as a function of its initial frequency? We consider biofilms comprised of 1 secretor strain mixed with 1, 2 or 4 non-secretor strains (**Q** or **C**: **N**—1:1, 1:2, and 1:4) to study the effect of increasing evolutionary competition (i.e. increasing local diversity). We also consider a range of quorum sensing strains (**Q1** to **Q4**) that all produce autoinducers at the same, constant rate, but are induced to produce public goods at different, increasing, threshold concentrations of the quorum sensing autoinducer.

**Fig 3:**
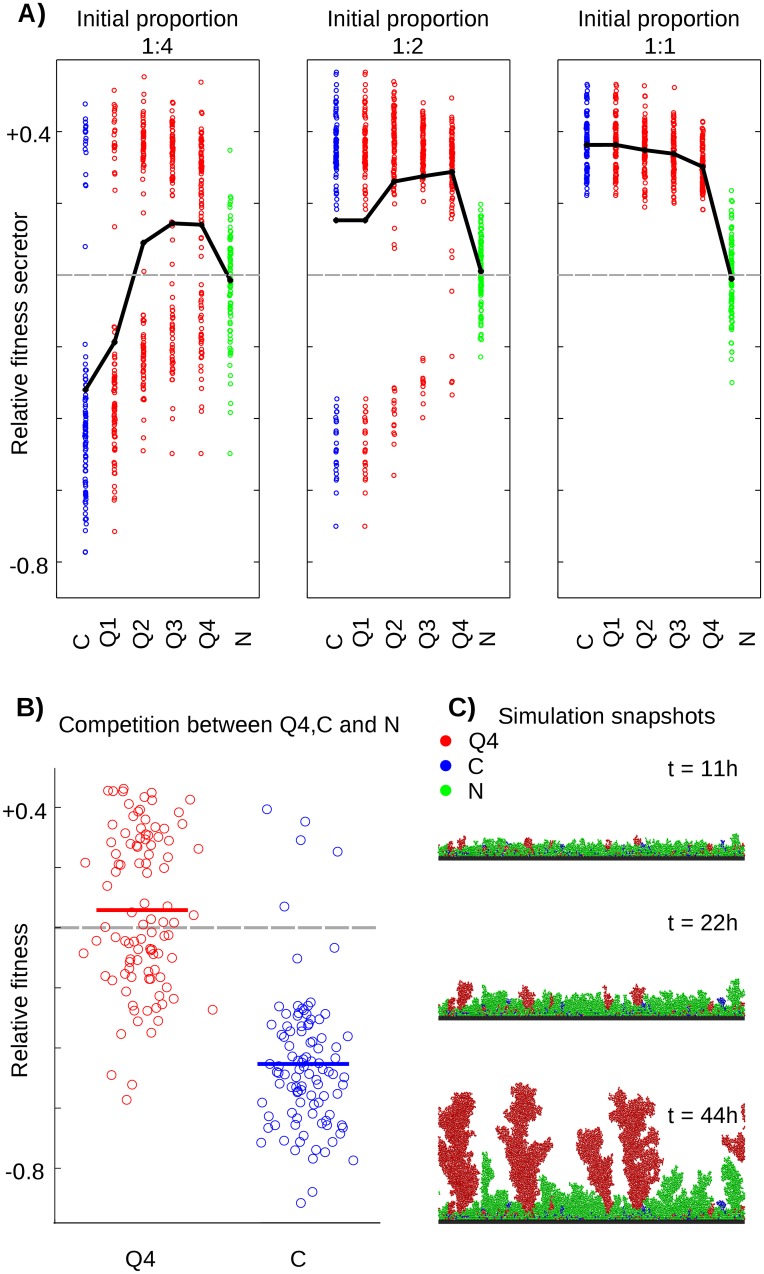
Quorum Sensing is beneficial in competitive, spatially structured environments. A) Relative fitness of different secretor genotypes (**C**: constitutive, **Q1–Q4**: quorum sensing secretors with increasing quorum sensing thresholds, see S3 Fig, **N**: non-secretor control) in competition with non-secretor genotypes. Higher genotypic diversity is reflected in lower initial proportions of the secretor genotype. We show results of 100 independent simulations each and plot the resulting mean relative fitness in black. **Q** genotypes have a higher relative fitness than **C** because they outgrow **N** more frequently (more runs fall above the 0 line in the bimodal distribution). This—rather than a higher benefit from the public good—is what allows **Q** to succeed, and is a result of **Q** genotypes competing better than **C** during the early stages of colony growth. B) Direct competition between **C**, **Q4** and **N**. 80 cells of **C** and **Q4** respectively, and 640 cells of **N** were seeded simultaneously. Relative fitness from 100 independent simulations was calculated for **Q4** and **C** by considering their fitness against the average competitor fitness (**N**+**Q4** and **N**+**C** respectively, see Methods), and the mean values are indicated by bars. C) Snapshots of an individual simulation shown in panel B where **Q4** succeeds.

When secretors (**C** or **Q1**–**4**) were seeded at a 1:1 proportion (low evolutionary competition), all five strains outcompete the **N** strain in each of the 100 simulations, with **C** achieving the highest mean relative fitness, although secretors do not differ much in their relative fitness (Fig 3A). This changes when we consider strong evolutionary competition with a low initial proportion of secretors. Importantly, **Q** strains succeed against competing non-secretors where constitutive secretors fail. Quorum sensing then enables cooperation in highly competitive environments where non-secretors would otherwise dominate. What is the cause of the advantage to **Q** strains over **C** strains under these conditions? Regulating secretion means that **Q** strains invest less in secretion overall than constitutive secretors. But in the supplement we reduce the investment of constitutive secretors to remove this difference and show that they still lose where **Q** strains win (S1 Fig). The key to success of **Q** then is the timing of secretion (in S2 Fig an artificial time-delay strategy demonstrates this). Quorum sensing allows a newly colonizing strain to compete against non-secretors and establish a clonal patch prior to activating energetically costly secretions, whereas **C** strains get overgrown and smothered while inefficiently investing into cooperative secretions. In these simulations of competitive, surface-attached communities then, the critical benefit to quorum sensing comes from the fact that expressing a costly secretion early means that such a genotype will be rapidly overgrown by faster growing genotypes.

### Quorum sensing as a mechanism to infer kinship

The benefit to quorum sensing that we observe is not directly linked to the assessment of cell density or total biomass. Rather, the relevant variable is the extent to which cells of a focal genotype are surrounded by clonemates relative to other cell types, which we compute using the “segregation index”. This segregation index is proportional to measures of kinship or relatedness within the range of a social trait [16, 33, 38] (Materials and Methods). A strain is predicted to be more successful then, if it can match its cooperative secretions not to the cell density or total biomass, but to the appropriate level of local relative cell density or genetic relatedness. Furthermore, as cells in mixed microbial colonies divide and increase in number, the likelihood of being surrounded by clonemates compared to different genotypes increases and average relatedness between neighbouring cells increases [30–32, 51, 52]; relatedness, then, correlates positively with time.

**Fig 4:**
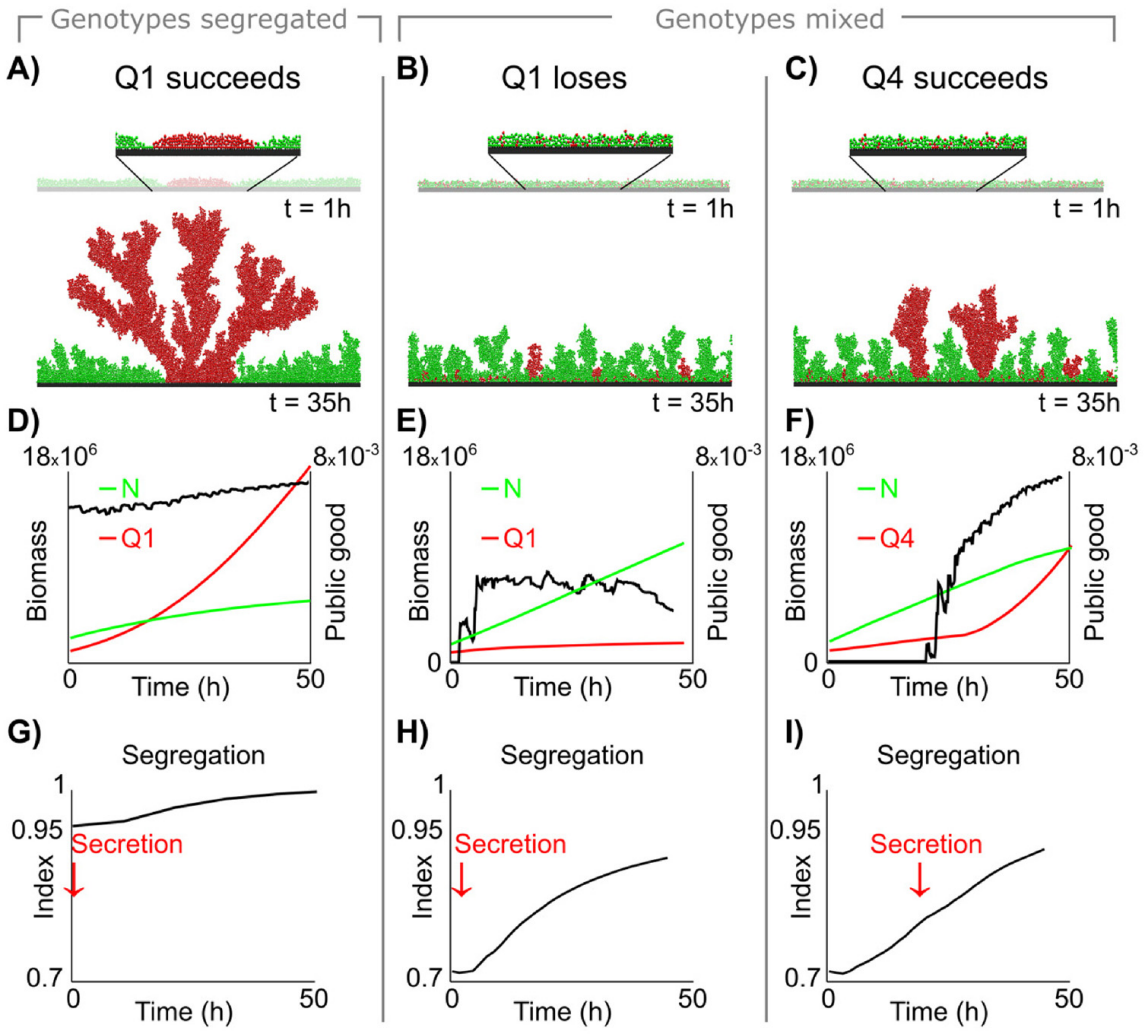
Autoinducer concentration tracks the shift from genetically-mixed to clonal groups. A) Snapshots of our simulations show how **Q1** (red) outompetes **N** (green) when cells are sorted by genotype (high relatedness from the start). Here, **Q1** (and even **C**, see S3 Fig) can outcompete **N**. **Q1** initiates public good production early while **Q4** delays secretion for longer due to its higher quorum sensing threshold (S1 Table). B) and C) show results from the same simulation setup but where cells were positioned randomly at the start (as in simulations in Figs 2 and 3). Now, **Q1** (red) cannot outcompete non-secretors (green) in local competition (B), whereas in C) **Q4** (red) outcompetes non-secretors (green). D-F) Left y-axis and red/green lines: Biomass over time of non-secretors **N** and secretor genotypes **Q1** and **Q4** in the corresponding simulations shown in A-C). Right y-axis and black lines: peak concentration of public good (maximum concentration in anywhere within the colony) in the biofilm over time. G-I) Segregation index which is a measure of relatedness in the simulations over time. Key to a successful strategy is initiating public good secretion only at a sufficiently high segregation index: when cells are sorted from the beginning, secretion can start early (A, G), but if cells are initially well-mixed, only delayed public good secretion leads to success (C, I).

The importance of relatedness over cell density in defining the quorum sensing threshold is demonstrated by Fig 4. We first consider a case where the seeding population is artificially sorted by genotype (**Q1** vs **N**) and population bottleneck effects are removed. This manipulation artificially increases relatedness from the start. Here a **Q1** genotype, which has a low quorum threshold and activates secretions immediately (or even a **C** genotype, S3 Fig), can outcompete non-secretors because they are already surrounded by many clonemates (Fig 4A). However, **Q1** (or **C**, S3 Fig) cannot outcompete non-secretors when we seed the same density of cells and genotypes but without forced sorting of genotypes (Fig 4B). Here, only a genotype with a higher quorum threshold (**Q4**) can outcompete non-secretors (Fig 4C).

**Q4** turns on public good production at a 4 times higher autoinducer concentration than **Q1**, which means that **Q4** delays costly secretion further in time (see S1 Table). In competition with the **N** genotype, **Q1** now gets buried under conditions where **Q4** can escape burial and form clonal towers. The reason is that **Q4** only initiates public good secretion when cells are surrounded by clonemates rather than others (Fig 4E and 4F) thereby amplifying the fitness gain from forming clonal clusters and outperforming non-secretors **N** on average (Fig 3). While still correlated with cell density, autoinducer concentration is tracking the shift from competitive, genetically-mixed populations to conditions where cells are surrounded by clonemates. Quantitatively, this can be seen from the fact that **Q4** starts secretion at a higher segregation index (Fig 4G–4I, Materials and Methods). And while other quorum sensing thresholds may initiate public good secretion at a more optimal moment compared to **Q4**, this example strategy illustrates that using a quorum sensing threshold to delay cooperation in dense and diverse communities can be advantageous. The optimal threshold will depend not only on cell density but, critically, on the ability to keep track of time and the concomitant change in local genetic relatedness, as the cell group transitions from low relatedness to sufficiently high relatedness under which cooperation becomes favourable.

Our model shows how a rare quorum sensing strain can succeed in a population of non-secretors, and so quorum sensing is predicted to evolve under these conditions. However, once common, how robust is quorum sensing-controlled public good secretion to potential exploitation by rare non-secretors? In the supplement we ask how a rare non-secretor genotype fares in local competition with **Q** (S4 Fig), which shows that quorum sensing strains also outcompete non-secretors when the latter are rare.

**Fig 5:**
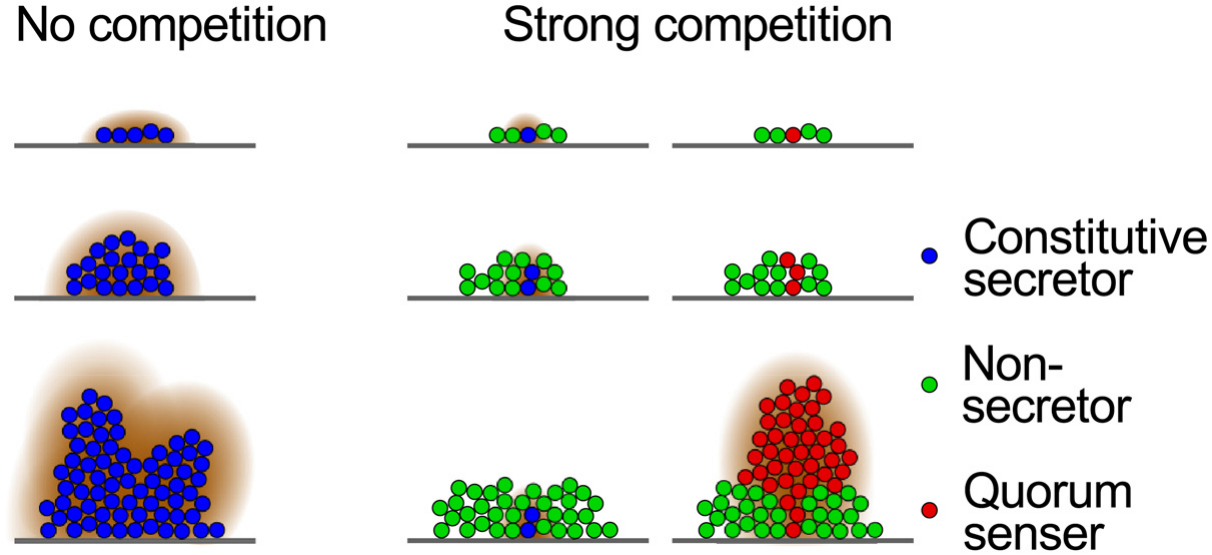
The benefit of quorum sensing under strong competition. Cartoon showing how quorum sensing genotypes can outperform constitutive secretors and non-secretors via its function as a timing mechansim. While constitutive secretors get overgrown by non-secretors, the delayed secretion of quorum sensing genotypes allows those cells to prevent being overgrown, by competing well during early stages, and as a result surrounding themselves with clonemates. Only then do they invest into a costly secretion.

Overall, our results show how the regulation of public good secretion by quorum sensing can be a more robust strategy than constitutive secretion in competitive environments. Specifically, in our system, quorum sensing strains can outcompete non-secretors where constitutive secretors will not (Fig 5). This is because quorum sensing strains can infer the local relative density of their own kin, and start cooperating once they have reached the appropriate level of genetic relatedness. But what about direct competition between a quorum sensing and a constitutive-secretor genotype? In general, these two genotypes perform similarly in direct competitions although quorum sensing genotypes do maintain a slight advantage (S5 Fig). This is again because **Q** cells grow faster initially and compete well for bottlenecks and, after establishing a clonal group, **Q** can benefit from its public good secretion. This initial edge leads to the success of **Q** over **C**, which is robust to reciprocal competition where a rare genotype **C** competes with frequent cells of genotype **Q**. The dominance of quorum sensing can also be seen in a competition involving all three main genotypes **C**, **Q** and **N** (Fig 3B and 3C), and in the supplement we show that the benefits of quorum sensing are robust to a more complex model where cells in different biofilms disperse and compete globally (S6 and S7 Figs).

### The benefit of quorum sensing does not rely on kin discrimination

In our model, only quorum sensing cells respond to autoinducers. Importantly, we also assumed that only the quorum sensing strains produce the autoinducer. This scenario will occur whenever quorum sensing strains differ from competing strains at both the loci for induction and response. The use of a genotype-specific signal, as observed in strains of *Bacillus*[47], raises the possibility that a focal genotype can detect the number of clonemate cells in the face of high variability in genetic mixing (a form of kin discrimination [55]). However, such kin discrimination is not needed for the evolutionary benefits to quorum sensing that we observe. In some situations, all genotypes may produce autoinducers even if they do not all respond to them, such as when autoinducers are linked to common metabolic waste products [54, 56]. We therefore also consider a system where all competing genotypes make the autoinducer. Under these conditions, **Q** cells maintain their advantage against non-secretor strains, albeit at different (higher) quorum sensing thresholds (S8 Fig).

While a genotype-specific signal is likely to be more robust to variability in strain mixing, therefore, it is not required for the evolutionary benefits to quorum sensing that we observe. Even if the change in autoinducer concentration reflects total cell density, a **Q** genotype can still use autoinducer concentration as a timer to infer genetic similarity. If the physical and social environment is sufficiently predictable, then, the indirect inference of kinship via autoinducers is sufficient for the effects we see, as opposed to strict kin detection and discrimination. This inference is possible whenever cell density correlates with the genetic similarity of neighbouring cells due to the changing spatial structure of the colony over time (Fig 4).

## Discussion

Two key issues have been identified as central to the evolution of quorum sensing to regulate the secretion of public goods. The first is a benefit over constitutive secretion. This is typically thought to come from the ability to infer cell density and perhaps diffusion rates. The second issue is that quorum sensing cells must resist competition from non-secreting cheater mutants. Here we show that, under conditions of strong competition, these two issues combine to provide a critical benefit to quorum sensing that is not simply due to the assessment of cell density. With nutrient limitation and high numbers of competing genotypes, the key benefit to quorum sensing in our system comes from the ability to delay public good secretion and grow quickly when first in a new environment. This allows quorum sensing cells to outgrow constitutive secretors while keeping up with fast growing non-secretors until clonal patches have formed in a microbial group. In particular, the delay in cooperative secretion in quorum sensing genotypes increases their chances of forming clonal clusters compared with constitutive secretors, and outcompeting them. Positive feedback from cooperation then amplifies the gain from forming a clonal cluster, which allows quorum sensing genotypes to outcompete non-secretors as well (Fig 5).

Our model makes some simplifying assumptions. Firstly, auto-inducer production carries no cost in our simulations. While this may not always be the case in reality, many quorum sensing molecules, such as autoinducer-2 (AI-2) carry little to no cost [57, 58]. Secondly, some modeling choices and parameter values, such as the quorum sensing thresholds, were determined based on previous work or by parameter sweeps rather than experimentally measured values. This approach then demonstrates that wide and realistic parameter ranges exist in which quorum sensing is adaptive. Finally, the importance of the predicted effect, whereby quorum sensing can delay cooperation until relatedness has increased will, of course, depend on how often microbes find themselves in highly competitive environments, which transition from diverse mixtures of strains to clonal clusters as they grow. In cases where cells grow in isolation as microcolonies, the benefits of quorum sensing are likely to come from the inference of cell density and diffusion as is typically assumed [10, 12, 13, 16]. However, there is a growing recognition of the importance of competition for understanding microbial phenotypes [5, 6, 54]. A focal microbial genotype may often land in a dense community where other genotypes are already present; in fact, most of microbial life is assumed to take place under these conditions [20]. And although our model here assumes that competing genotypes differ only in a few loci with all else being equal, higher background diversity will not alter our conclusions. Our model suggests then that quorum sensing can be particularly advantageous when competition between many different genotypes is fierce. This leads to a testable hypothesis: when surrounded by foreign genotypes, a strain that uses quorum sensing to control public good secretion can grow as fast as non-secretors and only activate secretions once it is safely surrounded by its own genotype.

## Materials and Methods

We are extending an individual based simulation framework developed and tested over the last 15 years [28, 59–61]. Cells are modelled individually and diffusion and reaction of chemical species are calculated by solving the reaction-diffusion equations. Briefly, cells take up and secrete chemical species, grow and divide once a certain threshold size is reached. Growth and division leads to overlapping cells. This overlap is relaxed by moving cells individually which leads to an expansion of the biofilm. There is no active movement and this relaxation mechanism is the only way cells can change their positions. Further, the substratum on which cells grow is impenetrable and cells cannot move into this area. We assume that reaction and diffusion of solutes happen on a much faster time scale than cell growth, division and biofilm expansion. The solute concentrations are, therefore, the steady state concentrations of the reaction-diffusion system for any particular biofilm conformation as in previous models [28, 61]. The framework is written in the Java programming language and the reaction-diffusion system is solved numerically to steady state with the relaxation method. For “well-mixed” simulations, cell positions were shuffled after each iteration and secretions such as autoinducers and extracellular secreted products were allowed to accumulate in the system by imposing dynamically changing boundary condition.

Here we adopt the same basic conventions and parameters as used in previous work on the evolution of public good secretion in biofilms [33, 38]. We extended them to include quorum sensing genotypes. The biofilm is represented in 2D. Cells grow on top of a hard, smooth surface that does not absorb or release any solutes. Above the biofilm, a diffusion layer separates cells from a region with constant nutrient concentrations (0.125 g/L, if not otherwise stated) and we impose periodic boundary conditions on the sides of the simulation area.

Our simulations include three types of cells: non-secretors (**N**), constitutive secretors (**C**) and quorum sensors (**Q**). **C** have exactly the same properties as **N** except that they divert a constant fraction of the nutrient uptake to the secretion of public good (here 20%). When the local public good concentration exceeds a threshold, the yield (i.e. cell growth per nutrient invested) increases by a factor of 3. This increase is the same for all three genotypes, i.e. all genotypes can benefit from the public good. **Q** are identical to **C** with the difference that they constantly secrete costless autoinducer molecules and only spend energy on public good production if the local auto-inducer concentration exceeds a certain threshold. This threshold varies for different types of **Q** genotypes. The exact rates and stoichiometry of all these processes can be found in S2 Table. The description and numeric value of all parameters are shown in S1 Table.

In each simulation a chosen number of cells of each genotype were seeded at random positions on the bottom surface. The cells were then allowed to grow as described above. The simulation was terminated once a fixed amount of nutrients had been absorbed by the biofilm and the final biomass was recorded for each genotype.

### Calculating fitness

We define fitness of each genotype (for example, *x* and *y*) as the mean number of rounds of cell division per unit time that cells of a focal genotype achieve during the interval between initial seeding at *t*_0_ and *t*_*end*_ when a maximum amount of nutrients were consumed. Fitness *w*, therefore, is calculated as

$$w_{x}=\frac{1}{t_{end}}log_{2}\frac{N_{x,t_{end}}}{N_{x,0}},$$ (1)

where *N*_*x*, *t*_ is the number of cells of genotype *x* present within the cell group at time *t*. The relative fitness of a genotype *x* in local competition with another genotype *y* is defined as: $\log_{10}\left(\frac{w_{x}}{w_{y}}\right)$ and, therefore, competition is successful when *w* > 0. The mean was taken over 100 such simulations and we show convergence of the results in the supplement (S7 Fig).

### Invasion analysis

A simple meta-population analysis was conducted following the same approach as in [33]. This determines whether a rare mutant strain would succeed in a metapopulation of cell groups with reoccurring dispersal and colonisation events. We assume a very large number of cell groups where the great majority of groups are of a single dominant strain and only a small minority will contain the mutant. Under these conditions, a genotype *x* (rare mutant) can invade a meta- population of genotype *y* (majority resident) if the fitness of *x* in local competition with *y* is greater than the average fitness of the whole metapopulation, denoted 〈*w*_*y*_〉. For each invasion analysis, *w*_*x*_ was computed in 100 replicates of the simulations (with varying inoculation frequencies of the two genotypes and a total of 800 cells initially, see relevant figures). Because the great majority of cell groups in the meta population consist purely of the majority genotype y, 〈*w*_*y*_〉 is approximately the mean fitness of the majority genotype, *y*, when growing on its own. To calculate 〈*w*_*y*_〉, the mean of *w*_*y*_ over 100 simulations is computed, where the cells of genotype *x* inoculated initially are replaced with *y* cells and a mono-culture of the majority genotype is simulated. The invasion index *I*_*x* → *y*_ of a rare mutant *x* into a metapopulation with majority genotype *y* was calculated for each of the 100 replicates as

$$I_{x\to y}=\frac{w_{x}}{\left\langle w_{y}\right\rangle }.$$

Under the assumptions of our model, we conclude that *x* can invade a metapopulation of *y* when *I*_*x* → *y*_ > 1.

### Statistical analysis

Simulation results are from 100 independent replicates. Fitness data is non-normal and often bimodal distributed where the bimodality differs between simulations with different initial frequencies and/or initial cell densities meaning it is difficult to apply standard statistics. In some figures we show box plots and test the median fitness value with non-parametric sign tests. This is only an indicator as from an evolutionary perspective, the mean relative fitness is the determining parameter of evolutionary success. Therefore, we further conducted convergence analyses that show how the mean fitness converges after about 100 simulations (S7 Fig).

### Genetic relatedness and the segregation index

The segregation index used here is identical to that used in previous work [38]. To measure segregation in a population of *M* cells, we consider each cell *c*_*i*_, *i* = 1…*M* in the population and identify all other individuals within a distance of 10*μ*m. The *N* cells in this neighbourhood are indexed by *c*_*j*_, with *j* = 1…*N*. We define a genotypic identity function, *g*(*c*_*i*_):

$$g(c_{i})=\left\{\begin{array}{c} 0,c_{j}\;\text{is}\;\text{not}\;\text{the}\;\text{same}\;\text{genotype}\;\text{as}\;c_{i}\\ 1,c_{j}\;\text{is}\;\text{the}\;\text{same}\;\text{genotype}\;\text{as}\;c_{i} \end{array}\right.$$ (2)

Segregation with respect to a focal cell, *s*(*c*_*i*_), was calculated as the mean product of the *g* and *m* functions for every cell in its neighbourhood:

$$s\left(c_{i}\right)=\frac{1}{N}\sum_{j=1}^{N}g(c_{j})$$ (3)

Finally, we define the segregation index *σ* for the entire cell group as the mean value of *s*(*c*_*i*_) across the population of cells:

$$\sigma =\frac{1}{M}\sum_{i=1}^{M}s\left(c_{i}\right)$$ (4)

The segregation index measures the degree to which co-localised cells are clonally related to each other. Relatedness in social evolution is defined as the probability that two individuals are more genetically similar than the population average. The segregation index is equal to a form of the relatedness coefficient from social evolution theory under the following assumptions: (i) A cell expressing the cooperative phenotype equally benefits all other individuals within a 10 cell-length radius; and (ii) Cell groups are seeded randomly from a large population pool [33]. In this meta-population, the frequency of a given focal strain is constant and small. To seed our simulations, then, we sample a number of strains (between 2 and 5, one of which is our focal strain) randomly from this population. The likelihood of a cell of the focal strain interacting with its clone in the meta-population is negligibly small. The segregation index then computes the likelihood of the focal cell interacting (presence over a 10 cell-length radius—this radius is somewhat arbitrary, and was kept identical to previous studies) with its clone relative to the null expectation in the metapopulation (close to 0).

## Supporting Information

S1 Fig Relative fitness of secretor genotypes (C: constitutive, C*: constitutive with reduced investment, Q4: quorum sensing secretor) in competition with non-secretor genotypes (N) in spatially structured communities. **Q4** succeeds against **N** whereas **C** loses. We then calculated the average amount of energy invested in public good secretion by the quorum sensing genotype that delays secretion (**Q4**) and matched the secretion rate of C* to ensure identical total investments (*R*_*E*_ = 0.8123). This does not reverse the fate of constitutive secretors: C* is still outcompeted by non-secretors. This demonstrates that the advantage of quorum sensing is not due to less investment in cooperation. The simulations are initialised with a 1:4 initial proportion of secretors to non-secretors. The results shown are from 100 independent simulations for each condition, the respective mean relative fitness is indicated by bars, and *** indicate *p*-values >0.001 from two-sided sign tests against a median of zero.
(EPS)

S2 Fig Introducing a simple time-delay to genotype C public good secretion recapitulates Q strategy. To demonstrate that the success of the **Q** genotype is due to a time delay in public good secretion, we implement such a delay in the **C** genotype and call this new strategy **Q^T^** where T represents the duration of the delay before public good secretion is initiated. **Q^0^** is thus equivalent to **C**. We then compete **Q^T^** against **N** at a 1:4 initial proportion of secretors to non-secretors. Blue dots show the fitness of **Q^T^** relative to **N** in each of 100 runs, while red dots shown the mean fitness of those runs. The figure shows that delaying public good secretion by 20 hours is equivalent to the strategy **Q4** (compare with Fig 3A, left in the main text).
(EPS)

S3 Fig Supplementary figure to Fig 4 in the main text. Spatial mixing (genetic relatedness) determines success of C genotype. Left: Snapshots of our spatially structured simulations show how **C** (blue) outcompetes **N** (green) when cells are sorted by genotype (i.e. genetic relatedness is high from the start). Right: here cells were positioned randomly at the start. Now, **C** (blue) cannot outcompete non-secretors (green) in local competition, as **Q4** does in Fig 4C in the main text. Bottom: Biomass over time of non-secretors (**N**) and constitutive secretor genotypes (**C**) in the corresponding simulations above.
(EPS)

S4 Fig Relative fitness of a non-secretor genotype (N) in competition with secretor genotypes in spatially structured simulations. **C**: constitutive, **Q1–Q4**: quorum sensing secretors with increasing quorum sensing thresholds, see S1 Table; **N**: non-secretor control. Higher genotypic diversity is reflected in lower initial proportions of the non-secretor, results of 100 independent simulations each, mean relative fitness in black.
(EPS)

S5 Fig Competition between quorum sensing genotypes and constitutive secretors. Left: Relative fitness of a rare constitutive secretor (**C**) in competition with quorum sensing genotypes. Right: a rare quorum sensing genotype (**Q**) in competition with constitutive secretors. Individual simulation results and mean (bold) in circles. *p*-values from non-parametric two-sided sign tests against zero median. While the mean relative fitness of a rare **Q** genotype is only slightly above 0, we show convergence of the mean in our simulations in S7 Fig.
(EPS)

S6 Fig Pairwise invasiveness analysis in spatially structured simulations. A) Summary of all pairwise invasion analyses under high evolutionary competition (initial frequency of invading genotypes 0.2). Here, arrows denote the direction of invasion with the rare genotype at the origin of the arrow. I.e., + next to the arrow from quorum sensing genotypes (**Q**, red arrows) to **N** denotes that **Q** can invade a metapopulation of non-secretors (**N**). On the other hand, constitutive secretors cannot invade non-secretors (**C**, blue arrows). Both secretor genotypes, however, resist reciprocal invasion by a rare non-secretor (green arrows), see also S7 Fig. Further, **Q** resists invasion by **C** but can itself invade **C**. The data from the perspective of **Q** is shown in B). Mean invasiveness values of 100 (200 for **Q** → **C**) independent simulations that are >1 indicate successful invasion of a metapopulation by the rare genotype (two-sided sign tests against medians of 1, *: *p* < 0.05;**: *p* < 0.01;***: *p* < 0.0001). We verified that the mean of the invasion **Q** → **C** whose median is least significantly different from 1 converges to a value >1 and that, therefore, **Q** can invade **C** (S7 Fig).
(EPS)

S7 Fig Invasion analysis of competitions between quorum sensing genotypes, constitutive secretor genotypes, and non-secretors in spatially structured simulations. Invasiveness values >1 indicate that a focal genotype can invade a metapopulation of the competitors under high competition (0.2 initial frequency) or low competition (initial frequency of rare genotype: 1, this assumes that patches of genotypes grow separated from each other), results of 100 independent simulations, mean values connected by the red line. A-C) Pairwise reciprocal invasion analysis of constitutive secretor (**C**), quorum sensing genotypes (**Q**) and non-secretors (**N**) into metapopulations the other genotypes. **Q** can invade metapopulations of **N** and **C** while itself resisting invasion from both. Against **C**, the differences are small but significant. Some of the data is bimodally distributed meaning it is difficult to use standard statistics (we show the results of two-sided sign tests against medians of 1, *: *p* < 0.05; **: *p* < 0.01; ***: *p* < 0.0001). We, therefore, further show an example of a convergence plot of the mean invasiveness value of **Q4** invading **C** over 200 simulations in D). The rolling mean invasiveness shows that after about 100 simulations additional simulations do not have a strong effect and the mean converges to a value slightly above 1 (blue line). Inset: histogram of bootstrapped means (solid vertical line at 1.01) and confidence intervals (dashed vertical lines at 0.98 and 1.04) indicate good accuracy of the original simulation data (10^6^ resampling events).
(EPS)

S8 Fig Relative fitness of a secretor genotype in competition with non-secretor genotypes (N) when all genotypes produce quorum sensing in spatially structured simulations. **C** is constitutive, **N** is non-secretor control, and **Q4–6** are quorum sensing genotypes with increasing quorum sensing thresholds, see S1 Table. Simulations assume 1:4 proportions of secretor to non-secretor genotypes, results of 100 independent simulations each, mean relative fitness in red. While **C** cannot outcompete its **N** competitors, delaying public good secretion conveys a competitive advantage to **Q** genotypes.
(EPS)

S1 Table List of parameters and the values used in our simulation models. *M*_*E*_ represents mass of extracellular enzyme, *M*_*I*_ represents mass of extracellular inducer, *M*_*G*_ represents mass of growth substrate, *M*_*X*_ represents cell biomass, *L* represents length, and *T* represents time.
(PDF)

S2 Table Stoichiometry of cell metabolism used in the simulation models. (PDF)

## References

[1] Fuqua W C, Winans SC, Greenberg EP (1994) Quorum sensing in bacteria: the LuxR-LuxI family of cell density-responsive transcriptional regulators. *J Bacteriol* 176:269–75. 8288518 10.1128/jb.176.2.269-275.1994 PMC205046

[2] Bassler B L (2002) Small talk: Cell-to-cell communication in bacteria. *Cell* 109:421–424. 10.1016/S0092-8674(02)00749-3 12086599

[3] Bassler B L, Losick R (2006) Bacterially speaking. *Cell* 125:237–246. 10.1016/j.cell.2006.04.001 16630813

[4] Diggle S P, Griffin A S, Campbell G S, West S A (2007) Cooperation and conflict in quorum-sensing bacterial populations. *Nature* 450:411–414. 10.1038/nature06279 18004383

[5] Cornforth D M, Foster K R (2013) Competition sensing: the social side of bacterial stress responses. *Nat Rev Microbiol* 11:285–293. 10.1038/nrmicro2977 23456045

[6] Mitri S, Foster K R (2013) The genotypic view of social interactions in microbial communities. *Annu Rev Genet* 47:247–273. 10.1146/annurev-genet-111212-133307 24016192

[7] Brown S P, Johnstone R A (2001) Cooperation in the dark: signalling and collective action in quorum-sensing bacteria. *Proc R Soc Lond B* 268:961–965. 10.1098/rspb.2001.1609 PMC1088694 11370970

[8] Rumbaugh K P, Trivedi U, Watters C, Burton-Chellew M N, Diggle S P, West S A (2012) Kin selection, quorum sensing and virulence in pathogenic bacteria. *Proc R Soc B* 279:3584–3588. 10.1098/rspb.2012.0843 22648154 PMC3396913

[9] Redfield R (2002) Is quorum sensing a side effect of diffusion sensing? *Trends Microbiol* 10:365–370. 10.1016/S0966-842X(02)02400-9 12160634

[10] Hense B, Kuttler C, Muller J, Rothballer M, Hartmann A, Kreft J-U (2007) Does efficiency sensing unify diffusion and quorum sensing? *Nat Rev Microbiol* 5:230–239. 10.1038/nrmicro1600 17304251

[11] Dulla G, Lindow S E (2008) Quorum size of *Pseudomonas syringae* is small and dictated by water availability on the leaf surface. *Proc Natl Acad Sci USA* 105:3082–3087. 10.1073/pnas.0711723105 18287070 PMC2268588

[12] Cornforth D M, Popat R, McNally L, Gurney J, Scott-Phillips T C, Ivens A, Diggle S P, Brown S P (2014) Combinatorial quorum sensing allows bacteria to resolve their social and physical environment. *Proc Natl Acad Sci USA* 111:4280–4284. 10.1073/pnas.1319175111 24594597 PMC3964068

[13] Brown S (1999) Cooperation and conflict in host-manipulating parasites *Proc R Soc Lond B* 266:1899–1904. 10.1098/rspb.1999.0864

[14] Sandoz K M, Mitzimberg S M, Schuster M (2007) Social cheating in Pseudomonas aeruginosa quorum sensing. *Proc Natl Acad Sci USA* 104:15876–15881. 10.1073/pnas.0705653104 17898171 PMC2000394

[15] Popat R, Crusz S A, Messina M, Williams P, West S A, Diggle S P (2012) Quorum-sensing and cheating in bacterial biofilms. *Proc R Soc B* 279:4765–4771. 10.1098/rspb.2012.1976 23034707 PMC3497100

[16] Cornforth D M, Sumpter D J T, Brown S P, Brännström A (2012) Synergy and group size in microbial cooperation. *Am Nat* 180:296–305. 10.1086/667193 22854073 PMC3635123

[17] Wilson D S (1975) A theory of group selection. *Proc Natl Acad Sci USA* 72:143–146. 10.1073/pnas.72.1.143 1054490 PMC432258

[18] Gans J, Wolinsky M, Dunbar J (2005) Computational improvements reveal great bacterial diversity and high metal toxicity in soil. *Science* 309:1387–1390. 10.1126/science.1112665 16123304

[19] Roesch L, Fulthorpe R, Riva A (2007) Pyrosequencing enumerates and contrasts soil microbial diversity. *ISME J* 1:283–290. 10.1038/ismej.2007.53 18043639 PMC2970868

[20] Lozupone C A, Knight R. (2007) Global patterns in bacterial diversity. *Proc Natl Acad Sci USA* 104:11436–11440. 10.1073/pnas.0611525104 17592124 PMC2040916

[21] Sekirov I, Russell S L, Antunes L C M, Finlay B B (2010) Gut microbiota in health and disease. *Physiol Rev* 90:859–904. 10.1152/physrev.00045.2009 20664075

[22] Dandekar A A, Chugani S, Greenberg E P (2012) Bacterial quorum sensing and metabolic incentives to cooperate. *Science* 338:264–266. 10.1126/science.1227289 23066081 PMC3587168

[23] Ben-Jacob E, Schochet O, Tenenbaum A, Cohen I, Czirók A, Vicsek T (1994) Generic modelling of cooperative growth patterns in bacterial colonies. *Nature* 368:46–9. 10.1038/368046a0 8107881

[24] Freese P D, Korolev K S, Jiménez J I, Chen I A (2014) Genetic drift suppresses bacterial conjugation in spatially structured populations. *Biophys J* 106:944–954. 10.1016/j.bpj.2014.01.012 24559997 PMC3944618

[25] Shapiro J A (1995) The significances of bacterial colony patterns. *BioEssays* 17:597–607. 10.1002/bies.950170706 7646482

[26] Habets M G J L, Rozen D E, Hoekstra R F, de Visser J A G M (2006) The effect of population structure on the adaptive radiation of microbial populations evolving in spatially structured environments. *Ecol Lett* 9:1041–8. 10.1111/j.1461-0248.2006.00955.x 16925653

[27] Hallatschek O, Nelson D R (2010) Life at the front of an expanding population. *Evolution* 64:193–206. 10.1111/j.1558-5646.2009.00809.x 19682067

[28] Xavier J B, Foster K R (2007) Cooperation and conflict in microbial biofilms. *Proc Natl Acad Sci USA* 104:876–881. 10.1073/pnas.0607651104 17210916 PMC1783407

[29] Mitri S, Clarke E, Foster K R (2015) Resource limitation drives spatial organization in microbial groups. *ISME J*. 10.1038/ismej.2015.208 26613343 PMC5029182

[30] Korolev K S, Müller M J I, Karahan N, Murray A W, Hallatschek O, Nelson D R (2012) Selective sweeps in growing microbial colonies. *Phys Biol* 9:026008 10.1088/1478-3975/9/2/026008 22476106 PMC3359763

[31] Golding I, Cohen I, Ben-Jacob E (1999) Studies of sector formation in expanding bacterial colonies. *Europhys Lett* 48:587–593. 10.1209/epl/i1999-00524-7

[32] Hallatschek O, Hersen P, Ramanathan S, Nelson D R (2007) Genetic drift at expanding frontiers promotes gene segregation. *Proc Natl Acad Sci USA* 104:19926–19930. 10.1073/pnas.0710150104 18056799 PMC2148399

[33] Nadell C D, Foster K R, Xavier J B (2010) Emergence of spatial structure in cell groups and the evolution of cooperation. *PLoS Comput Biol* 6:e1000716 10.1371/journal.pcbi.1000716 20333237 PMC2841614

[34] Van Dyken J D, Müller M J I, Mack K M L, Desai M M (2013) Spatial Population Expansion Promotes the Evolution of Cooperation in an Experimental Prisoner’s Dilemma. *Curr Biol* 23:919–923. 10.1016/j.cub.2013.04.026 23664975 PMC4405629

[35] Datta M S, Korolev K S, Cvijovic I, Dudley C, Gore J (2013) Range expansion promotes cooperation in an experimental microbial metapopulation. *Proc Natl Acad Sci USA* 110, 7354–9. 10.1073/pnas.1217517110 23569263 PMC3645579

[36] Nadell C D, Bucci V, Drescher K, Levin S A, Bassler B L, Xavier J B (2013) Cutting through the complexity of cell collectives. *Proc R Soc B* 280:20122770 10.1098/rspb.2012.2770 23363630 PMC3574390

[37] Kim W, Racimo F, Schluter J, Levi S B, Foster K R (2014) Importance of positioning for microbial evolution. *Proc Natl Acad Sci USA* 111:E1639–E1647. 10.1073/pnas.1323632111 24715732 PMC4000849

[38] Mitri S, Xavier J B, Foster K R (2011) Social evolution in multispecies biofilms. *Proc Natl Acad Sci USA* 108:10839–10846. 10.1073/pnas.1100292108 21690380 PMC3131810

[39] Schluter J, Nadell C D, Bassler B L, Foster K R (2014) Adhesion as a weapon in microbial competition. *ISME J* 9:139–149. 10.1038/ismej.2014.174 25290505 PMC4268496

[40] Cicmanec J F, Holder I A (1979) Growth of Pseudomonas aeruginosa in normal and burned skin extract: role of extracellular proteases. *Infect Immun* 25:477–483. 114486 10.1128/iai.25.2.477-483.1979 PMC414477

[41] Koschwanez J H, Foster K R, Murray A W (2011) Sucrose utilization in budding yeast as a model for the origin of undifferentiated multicellularity. *PLoS Biol* 9:e1001122 10.1371/journal.pbio.1001122 21857801 PMC3153487

[42] Darch S E, West S A, Winzer K, Diggle S P (2012) Density-dependent fitness benefits in quorum-sensing bacterial populations. *Proc Natl Acad Sci USA* 109:8259–8263. 10.1073/pnas.1118131109 22566647 PMC3361460

[43] Pai A, Tanouchi Y, You L (2012) Optimality and robustness in quorum sensing (QS)-mediated regulation of a costly public good enzyme. *Proc Natl Acad Sci USA* 109:19810–19815. 10.1073/pnas.1211072109 23144221 PMC3511743

[44] Salmond G P, Bycroft B W, Stewart G S, Williams P (1995) The bacterial’enigma’: cracking the code of cell-cell communication. *Mol Microbiol* 16:615–624. 10.1111/j.1365-2958.1995.tb02424.x 7476157

[45] Wang D, Ding X, Rather P N (2001) Indole Can Act as an Extracellular Signal in Escherichia coli. *J Bacteriol* 183:4210–4216. 10.1128/JB.183.14.4210-4216.2001 11418561 PMC95310

[46] Williams P, Winzer K, Chan W C, Cámara M (2007) Look who’s talking: communication and quorum sensing in the bacterial world. *Phil Trans R Soc B* (2007) 362:1119–1134. 10.1098/rstb.2007.2039 17360280 PMC2435577

[47] Stefanic P, Mandic-Mulec I (2009) Social interactions and distribution of Bacillus subtilis pherotypes at microscale. *J Bacteriol* 191:1756–1764. 10.1128/JB.01290-08 19114482 PMC2648371

[48] Lozupone C, Faust K, Raes J, Faith J J, Frank D N, Zaneveld J, Gordon J I, Knight R (2012) Identifying genomic and metabolic features that can underlie early successional and opportunistic lifestyles of human gut symbionts. *Genome Res* 22:1974–1984. 10.1101/gr.138198.112 22665442 PMC3460192

[49] West S A, Griffin A S, Gardner A, Diggle S P (2006) Social evolution theory for microorganisms. *Nat Rev Microbiol* 4:597–607. 10.1038/nrmicro1461 16845430

[50] Momeni B, Waite A J, Shou W (2013) Spatial self-organization favors heterotypic cooperation over cheating. *eLife* 2:1–18. 10.7554/eLife.00960 PMC3823188 24220506

[51] Nei M, Maruyama T, Chakraborty R (1975) The bottleneck effect and genetic variability in populations. *Evolution* 29:1–10. 10.2307/2407137 28563291

[52] Excoffier L, Ray N (2008) Surfing during population expansions promotes genetic revolutions and structuration. *Trends Ecol Evol* 23:347–351. 10.1016/j.tree.2008.04.004 18502536

[53] Hamilton W D (1964) The genetical evolution of social behaviour. I & II *J Theor Biol* 7:1–52. 10.1016/0022-5193(64)90038-4 5875341

[54] Hibbing M, Fuqua C (2009) Bacterial competition: surviving and thriving in the microbial jungle. *Nat Rev Microbiol* 8:15–25. 10.1038/nrmicro2259 PMC2879262 19946288

[55] Grafen A (1990) Do animals really recognize kin? *Anim Behav* 39:42–54. 10.1016/S0003-3472(05)80724-9

[56] Taga M E, Bassler B L (2003) Chemical communication among bacteria. *Proc Natl Acad Sci USA* 100 Suppl2:14549–14554. 10.1073/pnas.1934514100 12949263 PMC304117

[57] Keller L, Surette M (2006) Communication in bacteria: an ecological and evolutionary perspective. *Nat Rev Microbiol* 4:249–258. 10.1038/nrmicro1383 16501584

[58] Heurlier K, Dénervaud V, Haas D (2006) Impact of quorum sensing on fitness of Pseudomonas aeruginosa. *Int J Med Microbiol* 296:93–102. 10.1016/j.ijmm.2006.01.043 16503417

[59] Picioreanu C, van Loosdrecht M C M, Heijnen J J (1998) Mathematical modeling of biofilm structure with a hybrid differential-discrete cellular automaton approach. *Biotechnol Bioeng* 58:101–116. 10.1002/(SICI)1097-0290(19980405)58:1%3C101::AID-BIT11%3E3.0.CO;2-M 10099266

[60] Kreft J-U, Picioreanu C, Wimpenny J W, van Loosdrecht M C M (2001) Individual-based modelling of biofilms. *Microbiology* 147:2897–2912. 10.1099/00221287-147-11-2897 11700341

[61] Xavier J B, Picioreanu C, van Loosdrecht M C M (2005) A framework for multidimensional modelling of activity and structure of multispecies biofilms. *Environ Microbiol* 7:1085–1103. 10.1111/j.1462-2920.2005.00787.x 16011747

