# Supplementary material for: The Evolution of Quorum Sensing as a Mechanism to Infer Kinship
Source: PLoS Comput Biol. 2016 Apr 27;12(4):e1004848. doi: 10.1371/journal.pcbi.1004848 (PMC4847791; doi:10.1371/journal.pcbi.1004848)
Supplement: S1 Table — ME represents mass of extracellular enzyme, MI represents mass of extracellular inducer, MG represents mass of growth substrate, MX represents cell biomass, L represents length, and T represents time. (PDF) [file pcbi.1004848.s009.pdf]

Symbol	Description	Dimension	Value
μ_{max}	Maximum cell growth rate of genotype	T^{-1}	1
τ	Threshold public good concentration	$M_E L^{-3}$	4×10^{-3}
ϕ_j	Threshold autoinducer concentration for quorum sensing genotype j	$M_I L^{-3}$	$[0.02, 0.04, 0.06, 0.08, 0.16, 0.32]$
B	Growth factor increase due to the presence of public good at or above threshold concentration τ	dimensionless	3
Z	Growth factor decrease in growth rate of secretor genotypes due to the costly secretion of public goods	dimensionless	0.3
D_G	Growth substrate (nutrient) diffusivity	$L^2 T^{-1}$	4×10^4
D_E	Extracellular secreted product diffusivity	$L^2 T^{-1}$	3×10^5
D_I	Auto-inducer diffusivity	$L^2 T^{-1}$	3×10^5
E_{bulk}	Bulk concentration of extracellular product	$M_E L^{-3}$	0
$[E]$	Local concentration of extracellular product	$M_E L^{-3}$	n/a
I_{bulk}	Bulk concentration of autoinducer	$M_I L^{-3}$	0
$[I]$	Local concentration of autoinducer	$M_I L^{-3}$	n/a
G_{bulk}	Bulk concentration of growth substrate (nutrient)	$M_G L^{-3}$	0.125 unless stated otherwise
$[G]$	Local concentration of growth substrate (nutrient)	$M_G L^{-3}$	n/a
K_G	Half saturation constant for growth substrate concentration	$M_G L^{-3}$	3.5×10^{-5}
	Total nutrient consumption after which fitness was calculated	M_G	10^7
$N_{x,t}$	Number of cells of genotype x in a cell group at time t	dimensionless	n/a
R_E	Rate of secretion of extracellular product	$M_E M_X^{-1} T^{-1}$	1
w_x	Fitness of genotype x	T^{-1}	n/a
X_i	Concentration of biomass of genotype i	$M_X L^{-3}$	n/a
Y	Yield of biomass per substrate	$M_X M_G^{-1}$	0.5
