# Supplementary material for: The Evolution of Quorum Sensing as a Mechanism to Infer Kinship
Source: PLoS Comput Biol. 2016 Apr 27;12(4):e1004848. doi: 10.1371/journal.pcbi.1004848 (PMC4847791; doi:10.1371/journal.pcbi.1004848)
Supplement: S2 Table — (PDF) [file pcbi.1004848.s010.pdf]

	Solutes			Particulates			
**Reaction**	G	E	I	X_C	X_Q	X_N	**Rate Expression**
C growth	$-1/Y$			$1 + Bf([E])$			$\mu_{max_a} \frac{[G]}{[G]+K_G} X_C$
Q growth	$-1/Y$				$1 + Bf([E])$		$\mu_{max} \frac{[G]}{[G]+K_G} X_Q$
N growth	$-1/Y$					$1 + Bf([E])$	$\mu_{max} \frac{[G]}{[G]+K_G} X_N$
Autoinducer secreted by Q			1				X_Q
Product secreted by C		1		$-Z$			$R_E \mu_{max} \frac{[G]}{[G]+K_G} X_C$
Product secreted by Q		1			$-Z$		$R_E \mu_{max} \frac{[G]}{[G]+K_G} X_Q \theta[I]$

$f([E])$ is a function of local extracellular product concentration, $[E]$, $f([E]) = \begin{cases} 0, [E] < \tau \\ 1, [E] > \tau \end{cases}$

$\theta[I]$ is a function of local quorum sensing concentration, $[I]$, $\theta[I] = \begin{cases} 0, [I] < \phi_j \\ 1, [I] > \phi_j \end{cases}$

All other symbols are defined in Table S1.
